# 

**Published:** 1905-07-22

**Authors:** 


### NOTICE TO CORRESPONDENTS.

All MSS., Letters, Books for Review, and other matters intended for the Editor should be addressed The Editor, "The Hospital," The Hospital Buildings, 28 & 29 Southampton Street, Strand, W.C.

The Editor cannot undertake to return rejected MS., even when accompanied by stamped directed envelope.

**Notice to Advertisers and Subscribers.**

All Advertisements, Orders for Copies of The Hospital, and Business Communications should be addressed to **THE MANAGER (Not to the Editor),** The Hospital, 28 & 29 Southampton Street, Strand, London, W.C.

The Cost of Subscription, post free, is at follows:—Per week, 3½d.; per quarter, 3s. 3d.; per half-year, 6s. 6d.; per annum, 13s. Foreign and Colonial:—Per annum, 19s. 6d., payable, in all cases, in advance.

**NOTICE.—Vol. XXXVII. of "The Hospital" October 1904 to March 1905), in handsome cloth binding, will shortly be on sale at the office of this Journal, price 10s. 6d. Cases for binding the Numbers contained in this Volume 2s. Index to Vol. XXXVII., 3½d., post free.**

**The Monthly Part for July is now ready. Price 1s., by post, 1s. 3d.**

### BOOKS RECEIVED.

Great Eastern Railway Company.

"Tourist Guide to the Continent."

Wm. Heinemann.

"Physiological Economy in Nutrition." By Russell H. Chittenden, Ph.D.

John Bale, Sons, and Danielsson, Ltd.

"Adenoid Growths of the Naso-Pharynx." By G. A. Garry-Simpson, M.R.C.S.

"Squint." By Claud Worth, F.R.C.S.

Alden and Co., Limited, Oxford.

"The Health of the Nation." By J. Theodore Dodd, M.A.

Robert Gibson and Sons, Limited, Glasgow.

"Drugs, Dreams, Drams." By A Septuagenarian.

H. J. Glaisher.

"Anæsthetic Difficulties and How to Combat Them." By A. de Prenderville.

Rebman, Limited.

"Health and Disease in Relation to Marriage." Vol. II. By Senator and Kaminer.

Longmans, Green and Co.

"Diseases of the Anus and Rectum." By Goodsall and Miles.

Baillière, Tindall and Cox.

"Manual of Midwifery." By Hy. Jellett, M.D.

"Carcinoma of the Rectum." By F. Swinford Edwards, F.R.C.S.

"Handbook of Intestinal Surgery." By L. A. Bidwell, F.R.C.S.

Homœopathic Publishing Company.

"Homœopathy Explained." By J. H. Clarke, M.D.

"Low's Handbook to the Charities of London, 1905.

Elliot Stock.

"Hints for Infant Feeding." By Dr. Helen Sergeant.

T. Nelson and Sons.

"Hypatia." By Charles Kingsley.

David Nutt.

"The Burden of Demos, and other Verses." By Mary A Vialls.

The Scientific Press, Limited.

"Outlines of Routine in District Nursing." By M. Loane.

### MEDICAL APPOINTMENTS.

**E. Faulknor Ackery**, L.R.C.P.Lond., M.R.C.S.Eng., L.D.S., Dental Surgeon at the North-West London Hospital. **John Bramley Ball**, L.D.S.R.C.S.Eng., Dental House Surgeon to Guy's Hospital. **R. W. Burnet**, M.D.Aberd., F.R.C.P.Lond., Consulting Physician to the Great Northern Central Hospital. **Alfred Clark**, L.R.C.P & S.Edin., L.F.P.S.Glasg., Public Vaccinator for Ashburton, New Zealand. **J. C. Collins**, M.B., Ch.B.Dub., Medical Superintendent of the Northern Wairoa Hospital at Aratapu, New Zealand. **A. Douglas**, M.B., Ch.M.Edin., Port Health Officer at Oamaru, New Zealand. **Arthur Alan Forty**, L.D.S.R.C.S.Eng., Dental House Surgeon to Guy's Hospital. **George Joseph Alphonsus Hall**, L.R.C.P. & S.Irel., Public Vaccinator for Birmingham, New Zealand. **Frank Hewkley**, M.B.Durh., F.R.C.S.Eng., Honorary Physician to the St. Pancras and Northern Dispensary. **Herbert Stott Lindsay**, M.R.C.S., L.R.C.P.Lond., Health Officer for the Port of Whitianga and Public Vaccinator for Mercury Bay, New Zealand. **Walter Moray Shand**, M.B., Ch.B.N.Z., M.R.C.S.Eng., Public Vaccinator for Wellington, New Zealand. **H. E. Symes-Thompson**, M.D., M.R.C.P.Lond., Physician with charge of out-patients to the Great Northern Central Hospital. **George Henry Trenfield**, L.R.C.P.Lond., M.R.C.S., Surgeon to the Midland Railway at Bristol. **Rupert Waterhouse**, M.D.Lond., M.R.C.S.Eng., Pathologist and Curator of the Museum at the Royal United Hospital, Bath. **Otho F. Wyer**, M.D.Glasg., Consulting Physician to the Warneford General Hospital, Leamington.

### A HANDBOOK FOR NURSES.

By Dr. J. K. WATSON. 5/- net., 5/4 post free.

A complete Handbook of Medical and Surgical Nursing by an experienced teacher. Dr. Watson's Handbook is admittedly the best work on Nursing yet issued. The new addition of Examination Questions on Nursing should prove valuable to Matrons and those engaged in instructing Probationers.

### PRACTICAL GUIDE TO SURGICAL BANDAGING AND DRESSINGS.

By WM. JOHNSON SMITH, F.R.C.S. 2/- post free.

A useful guide to Bandaging and Dressing, written especially for Nurses by a surgeon of great experience. The work is up to date and the information thoroughly reliable.

### THE NURSES' PRONOUNCING DICTIONARY OF MEDICAL TERMS AND NURSING TREATMENT.

By HONNOR MORTEN. 2/- post free.

This Dictionary has been used by Nurses for many years, and the fact that it has been recently revised and the phonetic pronunciations added by Miss M. I. Burdett should greatly enhance its value as a work of reference. Its size enables it to be carried comfortably in the apron pocket, which is a strong point in its favour.

### A COMPLETE HANDBOOK OF MIDWIFERY.

By Dr. J. K. WATSON,
Author of "A Handbook for Nurses."
6/- net, 6/4 post free.

This work contains within its pages all that a midwife requires to know on the subject treated. It is written especially to meet the requirements of the Central Midwives' Board, and is well illustrated with some 143 diagrams.

### ELEMENTARY TREATISE ON THE LIGHT TREATMENT FOR NURSES.

By JAMES H. SEQUEIRA, M.D. Lond.
2/6 net, 2/9 post free.

As this treatment is becoming more generally practised, it has become essential that the details of the subject should be thoroughly understood by Nurses. The experience Dr. Sequeira has acquired in this special department of the London Hospital entitles him to speak with authority on the subject.

### THE MODERN NURSING OF CONSUMPTION.

By JANE H. WALKER. 1/2 post free.

The author has had considerable experience of the "Open-air" treatment of Consumption, and Nurses will find the work of great help in acquiring the principles underlying the modern treatment of this disease.

**The Scientific Press, Ltd.**

THE NURSERY OF NURSING TEXT-BOOKS,
28 & 29 Southampton Street,
Strand, London, W.C.

Complete Catalogue

Post free on application.

### The NURSING MIRROR.

**The Nurse's Own Paper.**

**Price ONE PENNY Weekly.**

Of all Booksellers and Newsagents, or posted direct from the Office.

*Write for Subscription Rates to The Manager,*

THE NURSING MIRROR, The Hospital Building, 28 & 29 Southampton Street, Strand, London, W.C.

